# Implante Transcateter de Valva Aórtica: O Que já Aconteceu e o que Ainda está Por Vir

**DOI:** 10.36660/abc.20230401

**Published:** 2023-07-21

**Authors:** David Costa de Souza Le Bihan, Rodrigo Bellio de Mattos Barretto, Wilson Mathias

**Affiliations:** 1 Hospital das Clínicas Faculdade de Medicina Universidade de São Paulo São Paulo SP Brasil Instituto do Coração do Hospital das Clínicas da Faculdade de Medicina da Universidade de São Paulo, São Paulo, SP – Brasil; 2 Fleury Medicina e Saúde São Paulo SP Brasil Fleury Medicina e Saúde, São Paulo, SP – Brasil

**Keywords:** Substituição da Valva Aórtica Transcateter, Implante de Valva aórtica Transcateter, Revisão Sistemática, Estenose da Válvula Aórtica

Em abril 2002 foi realizado o primeiro implante transcateter de valva aórtica (TAVI) em seres humanos, ainda como um procedimento experimental, em um paciente com estenose aórtica e choque cardiogênico, sem condições para realizar intervenção cirúrgica, tendo nesse procedimento compassivo a sua única possibilidade de sobreviver.^1^ Para chegar a esse momento, muitos esforços já tinham sido empregados no desenvolvimento de protótipos por quase 20 anos anteriores, mas provavelmente o Dr. Alain Cribier já tinha a ideia, naquela época do primeiro implante, da revolução que estaria por vir.^2^

Passados 21 anos do primeiro caso, o TAVI atingiu agora a maior idade. Hoje, faz parte da rotina do cardiologista clínico, que passou a confiar nele como uma real alternativa frente ao tratamento cirúrgico convencional. O TAVI revolucionou a forma como tratamos aquela que é a doença valvar mais prevalente em países desenvolvidos, atingindo até 7% da população acima de 65 anos.^2^

O TAVI trouxe consigo muitas novidades. Uma delas, foi a incorporação nos hospitais dos chamados “ *heart teams* ”, ou seja, grupos compostos por cardiologistas clínicos, intervencionistas e especialistas em imagens, além de cirurgiões cardiovasculares e anestesiologistas, que passaram a se reunir de forma sistemática para discutir casos clínicos, buscando a melhor alternativa para tratar um doente específico.^3^ Além disso, a partir do TAVI, o tratamento percutâneo das doenças estruturais do coração ganhou muito impulso e hoje falamos corriqueiramente de reparo *valve-in-valve, valve-in-mac* , implante percutâneo de prótese pulmonar, reparo mitral borda a borda, reparo tricúspide percutâneo, oclusão de refluxos paravalvares, entre outros procedimentos.

Ao longo desses anos, o TAVI se desenvolveu e mudou muito. As próteses evoluíram, tornando o implante mais fácil, com resultados mais previsíveis e com baixos índices de complicações. Além disso, algumas próteses passaram a apresentar capacidade de recuperação antes da liberação final e aumentaram as facilidades para acesso aos óstios coronarianos em intervenções futuras.^4^ Essas novas tecnologias, o treinamento das equipes e dos *heart teams* têm colaborado para a diminuição significativa das complicações associadas ao TAVI ao longo desses 21 anos.^5^

Os procedimentos também se tornaram mais simples, sendo realizados sob sedação, sem intubação traqueal e com monitoração apenas por ecocardiograma transtorácico.^6^ De fato, atualmente são realizados com anestesia geral e ecocardiograma transesofágico tridimensional apenas os casos com desafios anatômicos, disfunção ventricular esquerda ou impossibilidade de uso de contraste iodado por disfunção renal.^7^ Nos demais casos, o ecocardiograma transtorácico para diagnóstico e em sala para avaliar complicações e a tomografia computadorizada antes do procedimento (para definição da anatomia do complexo aórtico e das vias de acesso) são suficientes para dar suporte a essa intervenção. Essa estratégia minimalista, de fato, refletiu em substancial economia para o TAVI.^8^

A evolução do TAVI também permitiu conhecer melhor suas limitações, tais como os anéis muito pequenos ou muito grandes, a calcificação que se estende para a via de saída do ventrículo esquerdo, os distúrbios elétricos avançados, os seios de Valsalva estreitos, os óstios coronarianos de implante baixo, vias de acesso estreitas e as valvas bivalvulares.^9^ Apesar de nenhum desses se tratar de contraindicação absoluta, implicam em maiores riscos, que atualmente são bem conhecidos e podem ser ponderados pelo *heart team* para tomada de decisão, frente à condição clínica do paciente e à possibilidade de cirurgia convencional.

Com todo esse suporte de conhecimento, as recomendações internacionais e brasileira passaram a indicar o TAVI para pacientes com estenose aórtica em todo o espectro de risco operatório, dependendo da situação clínica.^10 - 12^ Dentro desse contexto, o artigo de Diegoli et al.,^13^ publicado nesta edição dos Arquivos Brasileiros de Cardiologia, compila os resultados dos grandes estudos randomizados que foram realizados.^13^ Ao reanalisar esses resultados em uma metanálise, chamam a atenção os melhores desfechos observados na população de risco cirúrgico baixo, para a qual a cirurgia convencional ainda é colocada como primeira opção de tratamento.^10 - 12^ A análise dos dados de Diegoli et al.^13^ nos desperta dúvidas sobre o futuro do tratamento para estenose aórtica: será o TAVI, em breve, a estratégia preferencial para todos os pacientes?^13^

A chave para essa resposta repousa sobre três pontos principais: a durabilidade das próteses percutâneas frente às próteses cirúrgicas, a presença de refluxos paraprotéticos (ainda mais frequentes no TAVI do que nas próteses cirúrgicas) e a necessidade de implante de marcapasso, também mais frequente no TAVI, como também demonstrado por Diegoli et al.,^13^ especialmente para as próteses autoexpansíveis.^14^

Quando se trata do primeiro ponto, estudos preliminares mostraram desfechos semelhantes quanto à degeneração estrutural, tanto para as próteses balão expansíveis quanto para as autoexpansíveis, quando comparadas às próteses cirúrgicas.^15 - 19^ Além disso, um estudo de custo efetividade mostrou discreta vantagem econômica para o TAVI, no acompanhamento em médio prazo.^16^ Entretanto, para pacientes mais jovens (abaixo de 70 anos), ainda não temos dados consistentes para a durabilidade das próteses TAVI frente às cirúrgicas.^10^

Em relação aos refluxos paraprotéticos, eles são infrequentes na cirurgia convencional. É fato que com as novas próteses TAVI sua incidência tem diminuído e, mesmo quando presentes, tendem a diminuir ao longo do tempo.^5 , 20^ Entretanto, sua presença é relevante e apresenta impacto negativo na sobrevida dos pacientes, mesmo para casos considerados discretos.^21 - 25^ Ainda, a necessidade de implante de marcapasso, que acontece em cerca de 17% nas próteses TAVI autoexpansíveis frente a 6% nas próteses cirúrgicas^26^ impacta nos custos médicos em longo prazo e na mortalidade dos pacientes após TAVI.^22 , 27 , 28^

Finalmente, não se conhece bem como pacientes com próteses aórticas percutâneas implantadas se comparam aos com próteses cirurgicamente implantadas, caso haja necessidade de reintervenção cirúrgica, visto que o explante das próteses percutâneas parece ser mais complexo e possivelmente envolve maior risco.

Portanto, apesar dos resultados animadores apresentados por Diegoli et al.,^13^ há necessidade de cautela. A “Odisseia do TAVI”^2^ ainda está acontecendo diante dos nossos olhos. Apesar dos excelentes resultados em curto prazo, ainda há muito o que estudar. A redução dos refluxos paraprotéticos e da incidência de marcapasso são desafios que estão à frente. A cirurgia convencional, que também apresentou grande evolução nos últimos anos, certamente tem seu espaço garantido na atualidade para os casos, não infrequentes, em que as limitações anatômicas tornam o TAVI um procedimento de maior risco, independentemente do perfil de paciente, bem como nas doenças multivalvares.
