# Supplementary material for: Implante Transcateter de Valva Aórtica: O Que já Aconteceu e o que Ainda está Por Vir
Source: Arq Bras Cardiol. 2023 Jul 21;120(7):e20230401. [Article in Portuguese] doi: 10.36660/abc.20230401 (PMC10421602; doi:10.36660/abc.20230401)
Supplement: Translation

# Translation: Transcatheter Aortic Valve Implantation: What has Happened and What is Yet to Come

**Authors:** David Costa de Souza Le Bihan, Rodrigo Bellio de Mattos Barretto, Wilson Mathias

**Affiliations:** 1 Brasil Instituto do Coração do Hospital das Clínicas da Faculdade de Medicina da Universidade de São Paulo, São Paulo, SP – Brasil; 2 Brasil Fleury Medicina e Saúde, São Paulo, SP – Brasil

**Keywords:** Transcatheter Aortic Valve Replacement, Transcatheter Aortic Valve Implantation, Systematic Review, Aortic Valve Stenosis

In April 2002, the first transcatheter aortic valve implantation (TAVI) was performed in humans, still as an experimental procedure, in a patient with aortic stenosis and cardiogenic shock, unable to undergo surgical intervention, with this compassionate procedure being his only possibility to survive.^1^ To get to this moment, much effort had already been taken into developing prototypes for almost 20 years previous, but probably Dr. Alain Cribier already had the idea, at that time of the first implant, of the revolution to come.^2^

After 21 years of the first case, TAVI has now come of age. Today, it is part of the routine of clinical cardiologists, who have come to trust it as a real alternative to conventional surgical treatment. TAVI has revolutionized how we treat the most prevalent valve disease in developed countries, affecting up to 7% of the population over 65.^2^

TAVI brought with it many novelties. One of them was the incorporation into hospitals of the so-called “heart teams,” that is, groups composed of clinical cardiologists, interventionists, and imaging specialists, in addition to cardiovascular surgeons and anesthesiologists, who began to meet systematically to discuss clinical cases, looking for the best alternative to treat a specific patient.^3^ In addition, from TAVI, the percutaneous treatment of structural heart diseases gained much momentum, and today we talk routinely about valve-in-valve repair, valve-in-mac, percutaneous implantation of the pulmonary prosthesis, edge-to-edge mitral repair, percutaneous tricuspid valve repair, occlusion of paravalvular leaks, among other procedures.

Over these years, TAVI has developed and changed a lot. Prostheses have evolved, making implantation easier, with more predictable results and low complication rates. In addition, some prostheses were manufactured with a recovery capacity before its final release, and with an easier access to the coronary ostia in future interventions.^4^ These new technologies, and the trainning of heart teams have contributed to a significant decrease in complications associated with TAVI over these 21 years.^5^

The procedures have also become simpler, being performed under conscious sedation, without tracheal intubation, and with monitoring only by transthoracic echocardiography.^6^ Currently, only cases with anatomical challenges, left ventricular dysfunction, or impossibility of using iodinated contrast due to renal dysfunction are performed with general anesthesia and three-dimensional transesophageal echocardiography.^7^ In other cases, transthoracic echocardiography (for diagnosis and during procedure, to evaluate complications) and computed tomography before the procedure (to define the anatomy of the aortic complex and the access route) are sufficient to support this intervention. This minimalist strategy resulted in substantial savings for TAVI.^8^

The evolution of TAVI has also made it possible to understand better its limitations, such as very small or very large annulus, calcification extending into the left ventricular outflow tract, advanced electrical disturbances, narrow sinuses of Valsalva, coronary ostia of low implant, narrow access ways and bicuspid valves.^9^ Although none of these are absolute contraindications, they imply greater risks, which are currently well known and can be considered by the heart team for decision-making, considering the clinical condition of the patient and the possibility of conventional surgery.

With all this background knowledge, international and Brazilian recommendations began to indicate TAVI for patients with aortic stenosis in the entire spectrum of operative risk, depending on the clinical situation.^10 - 12^ Within this context, the article of Diegoli et al.,^13^ published in this issue of *Arquivos Brasileiros de Cardiologia* , compiles results from large randomized trials that have been performed.^13^ When reanalyzing these data in a meta-analysis, they observed better outcomes in the low-surgical-risk population, for whom conventional surgery is still considered the first treatment option.^10 - 12^ The data analysis by Diegoli et al.^13^ raises questions about the future of treatment for aortic stenosis: will TAVI soon be the preferred strategy for all patients?^13^

The key to this answer rests on three main points: the durability of percutaneous prostheses compared to surgical prostheses, the presence of paraprosthetic leaks (still more frequent in TAVI than in surgical prostheses), and the need for pacemaker implantation, also more frequent in TAVI, as also demonstrated by Diegoli et al.,^13^ especially for self-expanding prostheses.^14^

Regarding the first point, preliminary studies have shown similar outcomes in terms of structural degeneration, both for balloon-expandable and self-expandable prostheses, when compared to surgical prostheses.^15 - 19^ In addition, a cost-effectiveness study showed a slight economic advantage for TAVI in the medium-term follow-up.^16^ However, for younger patients (below 70 years), we still do not have consistent data for the durability of TAVI prostheses compared to surgical prostheses.^10^

Regarding paraprosthetic leaks, they are infrequent in conventional surgery. It is a fact that with the new TAVI prostheses, their incidence has decreased; besides, even when present, they tend to decrease over time.^5 , 20^ However, its presence is relevant and has a negative impact on patient survival, even for cases considered as a mild regurgitation.^21 - 25^ Furthermore, the need for pacemaker implantation, which occurs in approximately 17% of self-expandable TAVI prostheses compared to 6% of surgical prostheses,^26^ impacts long-term medical costs and patient mortality after TAVI.^22 , 27 , 28^

Finally, it is not well known how patients with implanted percutaneous aortic prostheses compare to those with surgically implanted prostheses, in case there is a need for surgical reintervention since the explantation of percutaneous prostheses seems to be more complex and possibly involves greater risk.

Therefore, despite the encouraging results presented by Diegoli et al.,^13^ caution is needed. The “TAVI Odyssey”^2^ is still happening before our eyes. Despite the excellent short-term results, there is still much to study. Reducing paraprosthetic leaks and the incidence of pacemakers are challenges ahead. Conventional surgery, which has also shown a great evolution in recent years, certainly has its space guaranteed nowadays for cases, not infrequent, in which anatomical limitations make TAVI a higher-risk procedure, regardless of the patient’s profile, as well as in the multivalvular diseases.
